# Defect Detection in Aerospace Sandwich Composite Panels Using Conductive Thermography and Contact Sensors

**DOI:** 10.3390/s20226689

**Published:** 2020-11-23

**Authors:** David I. Gillespie, Andrew W. Hamilton, Robert C. Atkinson, Xavier Bellekens, Craig Michie, Ivan Andonovic, Christos Tachtatzis

**Affiliations:** 1 Department of Electronic and Electrical Engineering, University of Strathclyde, Royal College Building, 204 George Street, Glasgow G1 1XW, Scotland, UK; robert.atkinson@strath.ac.uk (R.C.A.); xavier.bellekens@strath.ac.uk (X.B.); c.michie@strath.ac.uk (C.M.); i.andonovic@strath.ac.uk (I.A.); christos.tachtatzis@strath.ac.uk (C.T.); 2 Collins Aerospace, Prestwick, 1 Dow Avenue, Prestwick International Aerospace Park, Ayrshire KA9 2SA, UK; 3 National Manufacturing Institute Scotland, University of Strathclyde, 85 Inchinnan Drive, Renfrewshire PA4 9LJ, UK; andrew.w.hamilton@strath.ac.uk

**Keywords:** aerospace composite, dis-bond, honeycomb, impact damage, maintenance repair overhaul, non destructive inspection, quality assurance, re-manufacture, sandwich structure, thermography

## Abstract

Sandwich panels consisting of two Carbon Fibre Reinforced Polymer (CFRP) outer skins and an aluminium honeycomb core are a common structure of surfaces on commercial aircraft due to the beneficial strength–weight ratio. Mechanical defects such as a crushed honeycomb core, dis-bonds and delaminations in the outer skins and in the core occur routinely under normal use and are repaired during aerospace Maintenance, Repair and Overhaul (MRO) processes. Current practices rely heavily on manual inspection where it is possible minor defects are not identified prior to primary repair and are only addressed after initial repairs intensify the defects due to thermal expansion during high temperature curing. This paper reports on the development and characterisation of a technique based on conductive thermography implemented using an array of single point temperature sensors mounted on one surface of the panel and the concomitant induced thermal profile generated by a thermal stimulus on the opposing surface to identify such defects. Defects are classified by analysing the differential conduction of thermal energy profiles across the surface of the panel. Results indicate that crushed core and impact damage are detectable using a stepped temperature profile of 80 ${}^{\circ }$C The method is amenable to integration within the existing drying cycle stage and reduces the costs of executing the overall process in terms of time-to-repair and manual effort.

## 1. Introduction

Aerospace Maintenance, Repair and Overhaul (MRO) services are critical to ensuring safe and certified commercial aircraft operation. The demand for these services in the UK alone accounts for an estimated £15 billion turnover annually and with the rapid increase in the number of commercial flights year-on-year, the value is set to increase [1]. For MRO service providers, lean innovation is central to ensuring that the capability in meeting the increased demand is satisfied through transitioning from traditional inspection methods based on manual processes with limited data capture towards digitised, sensor-based, data-driven methodologies [2,3].

A major structural component used for aircraft surfaces are sandwich panels comprising two outer skins of Carbon Fibre Reinforced Polymer (CFRP) and an inner core of aluminium honeycomb [4]. The composition has unique properties, with the skins providing protection against impacts and stiffness across the surfaces of the panel. The honeycomb core has low density but high strength in the through-thickness direction, with the skins preventing compression or tension spatially across the surface. The configuration is often used in aerospace structures subject to continual bending moments, the skins able to resist the compressive or tensile forces and the core acting as the neutral centre-line of the moment. The forces are concentrated on the surface skins which have much higher Young’s moduli, allowing the high strength CFRP to resist stresses. Whilst sandwich panels have desirable material properties, the structure is prone to a number of critical mechanical fault mechanisms [5,6].

### 1.1. Defects within Sandwich Panels

During routine aircraft service, a variety of objects can impact the surface, ranging from bird strikes to collisions with ground vehicles/equipment, creating damage to the sandwich structure. The mechanical damage to the panel is classified as:

- Dis-bond—the core and skin become detached over a significant spatial region of the panel as a consequence of the high shear stresses at the interface and the effect of externals impacts. Whilst the skin and core may remain in contact due to the surrounding panel remaining intact, discontinuity typically results in a thin void and a reduction in material strength.
- Delamination—a void can form within either of the sandwich skins owing to shear stresses or impacts; two or more layers of CFRP that compose the skin can separate. Similar to dis-bonds, a thin void will be present with concomitant loss in structural strength.
- Crushed Core—the aluminium honeycomb is compressed in one or more cells due to an external impact.

Whilst the above classes of mechanical defects can occur individually, the most common fault mechanism is through impacts on the component surface. Some impacts are obvious due to visible damage or surface warping and are readily identified for repair through visual inspection; however more challenging to identify are defects classed as Barely Visible Impact Damage (BVID) [7]. In the latter case, the impact leaves little or no evidence of damage upon the surface of the structure and can consist of one or a combination of dis-bonds, delaminations and crushed cores beneath the structure surface. BVID requires non-destructive inspection methods that detect subsurface defects.

A damaging non-mechanical defect that plagues sandwich panels is the ingress of oil and other liquid contaminants into the voids of the honeycomb core. A variety of engine oils, mechanical component lubricants and fuel will transfer from their intended locations into the voids within the cells of the honeycomb through flow paths during routine aircraft operation. The paths follow porous regions of the sandwich panel skin, or flow through mechanical defects. Operationally, oil ingress is difficult to prevent even with robust design and often, aircraft sandwich panel structures are removed for normal servicing to undergo ‘drying’ cycles. The component is heated to a sufficient temperature of 80 ${}^{\circ }$C within a vacuum for the oil contaminants to be drawn out into a porous collection material. Contaminants accumulate over a period of months, whilst the drying cycle is normally less than 2 h.

### 1.2. Repair of Defects

Once dis-bonds and delaminations have been identified, repairs consist of removing the damaged skin section with adjacent areas and replacing the skin and/or core with new material. The entire sandwich panel must be vacuum sealed and placed within Collins Aerospace’s 18 foot diameter, custom built AeroThermal group/AIC group, autoclave. The panel is then placed under a uniform pressure of 7 bar, applied via compressed Nitrogen gas, to the component surface. A ramped thermal profile is then applied according the composite material’s manufacture specifications (180 ${}^{\circ }$C). The material manufactures specifications for a thermal curing profile ensures curing of the CFRP without uncontrolled exothermic reactions damaging the material [8,9]. The pressure within the autoclave during curing prevents the formation of voids and expels moisture and volatiles within the CFRP [10,11,12]. Due to the high temperatures required within an autoclave curing cycle, it is possible for undetected defects such as delaminations and dis-bonds to propagate through variations in thermal expansion between the quality structure and structural area with defects [13,14]. Post the repair autoclave curing cycle, the component is re-inspected for damage and any minor defects which were previously undetected but have expanded into significant damage post repair curing also require repair through a further autoclave cycle. Consequently, MRO operators may process the same panel multiple times ‘chasing’ the defects. The cost of the local repair of a defect as opposed to an entire re-skinning (the fabrication and replacement of the entire damaged laminate structure) becomes unsustainable and the latter option is invariably adopted, resulting in a financial loss. An inspection method that offers full coverage of the component and able to identify all damage, empowers operators to decide if local repair of damage is economical when compared to the possibility of a full re-skinning after several cycles of chasing repairs.

### 1.3. Testing and Inspection in Aerospace MRO

Two types of strategies for detecting defects are adopted by the aerospace MRO industry; inspection and test [15,16]. Test processes are governed by stricter safety standards set by International Aviation Safety Authorities e.g., the European union Aviation Safety Agency (EASA) [17], the overarching organisation for safety compliance, provides oversight of approved national organisations within the European Union that monitor MRO service operators. The authorities define the acceptable methods for testing components considered critical to the safe operation of aircraft such as air-frame structures or engine mounting frames. Inspection processes are governed by a set of safety standards applicable to components considered to be less critical to the safe operation of aircraft e.g., sandwich panels used as protective surfaces of components. Whilst reducing the incidence of faults on all structures is paramount, a fault developing during flight within a component covered by inspection methods does not have a catastrophic impact.

### 1.4. Inspection of Sandwich Panels

Manual acoustic tap inspection is an industry standard method to identify dis-bonds, delaminations and crushed cores. A trained MRO operator will use a testing hammer to lightly tap the surface of the sandwich panel at regular intervals. The presence of defects under the surface produces a ‘duller’ tone than non-defective surfaces due to the dampening of the acoustic resonance of the sandwich structure [18]. The process is labour-intensive and relies on the experience of the operator to discern the difference between defective and normal regions. The location of potential defects are marked upon the structure itself for repair after the drying cycles have been completed. When compared to other inspection methods where a recording is taken at each point (e.g., the resonance of an ultrasonic wave when using an ultrasonic A-scan), the manual tap inspection method is unable to produce a detailed report other than the markings upon the structure surface. In regard of the requirement to maintain records of damage, this technique is limited to photographs, captured by commercial digital cameras to provide an approximate location of the damage and an approximate areal measurement in mm${}^{2}$ conducted by an operator manually measuring the area by means of a rule. These photographic records and basic areal measurements provide only a reference as to the history of damage on the component. Conductive thermography for sandwich panels is the analysis of the differential thermal transmission to determine the presence of defects [19,20,21]. The introduction of spatially uniform thermal energy into the inner (or outer) CFRP skin of the sandwich panel and monitoring the change in temperature across the surface of the opposing side can be used to detect defects. Damage to the core or the adhesive bond between the laminate and core introduce voids that reduce thermal conductivity at the macro-scale. The relationship can be described by in it’s simplest state through Fourier’s law of thermal conduction

(1) q=−kΔT

where *q* is heat flux, *k* is thermal conductivity and ΔT is the change in temperature between surfaces.

A homogeneous structure consisting of uniform material composition, such as an aluminium honeycomb CFRP sandwich structure, would display a constant *k* value. Any modification to the *k* within the structure implies that the area is not of the same composition as the surrounding homogeneous regions.

Whilst other non-destructive inspection methods (NDI) exist such as ultrasonic inspection, the main advantage of non Infrared (IR) conductive thermography is the potential to integrate the technique into the drying cycle phase. The heating source used to characterise the sandwich panel structure for defect detection can then be used to remove oil through reducing its viscosity under vacuum. The capability to detect defects and then apply the drying cycle using the same equipment would dramatically reduce inspection and panel processing times. Given the need to execute the process under vacuum, the line-of-sight required to conduct traditional IR thermography is obstructed with in-vacuum components such as bagging and breather material. It has been demonstrated that readily available equipment and temperature sensors which are compatible with the vacuum process with the MRO environment can identify laminate material properties of composites at low temperatures [22]. As recordings of temperature against time are taking at known locations using conductive thermography, this form of inspection allows for a detailed report to be generated and recorded. The paper presents a method of identifying structural defects by means of capturing areas of reduced thermal conductivity that does not require an unobstructed line-of-sight to the component, currently unavoidable with traditional IR thermography techniques.

## 2. Experimental Setup

**Figure 1:**
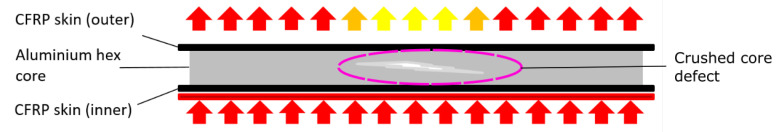
Transmission thermography basic principle of defect detection through detection of thermal transfer changes. Thermal energy shown in red arrows (**bottom**); the resultant thermal energy due to thermal conduction shown in the red to yellow arrows (**top**) displays a reduction in thermal energy over the defect area within the Aluminium core.

The paper reports on an alternative to non-IR conductive thermography, although based on the principles of the latter as it relies on the measurement of the rate of thermal transfer on the skin of a sandwich panel with uniform thickness and structural composition, due to the thermal energy stimulus applied on the opposing skin. The presence of defects modulates the rate of thermal transfer spatially (Figure 1) across the opposing skin. This method still requires a distributed method of monitoring temperature, however it is one that does not rely on line-of-sight methods such as IR cameras. A number of representative test panels were manufactured, inspected using the proposed approach and results analysed to confirm the potential of a non-IR conductive thermography technique to detect defects within aerospace sandwich panel structures.

### 2.1. Sample Sandwich Panels

**Figure 2:**
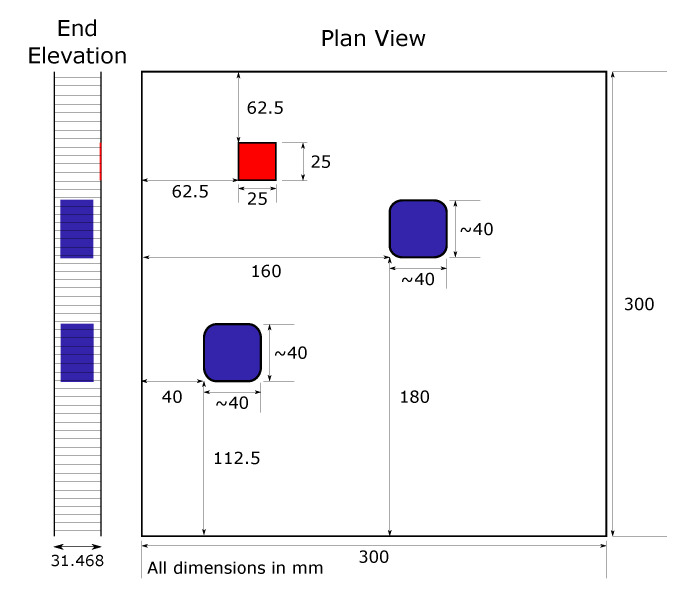
Panel D1 defect locations. Delamination in red, impact damage blue.

**Figure 3:**
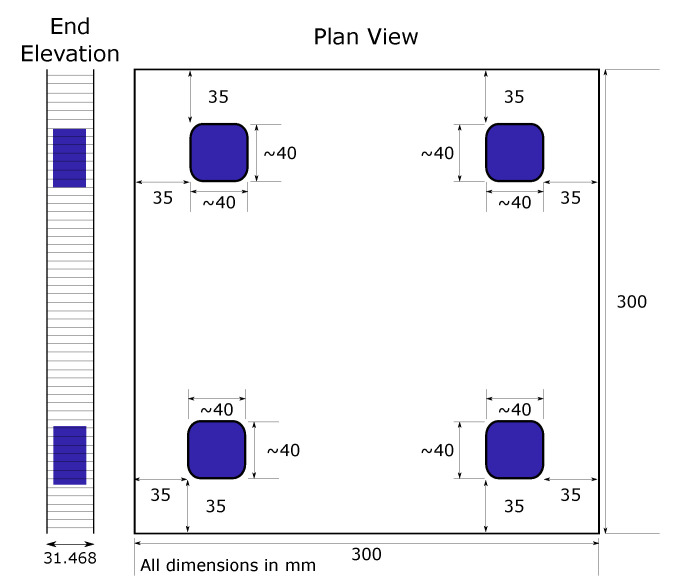
Panel D2 defect locations. Impact damage blue.

A set of test samples representative of the structure used for engine nacelle fan cowls were manufactured. The inner and outer skins comprise 0.734 mm thick CFRP laminates using 2 plies of a 5 harness bi-axial woven pre-impregnated. The skins were bonded to a 30 mm thick aluminium core with cell radius of 5 mm and cell aluminium skin of 1 mm. The characteristics of each panel were as follows;

- D0—No defects; the baseline sample to calibrate the control system and data acquisition prior to testing on defective panels.
- D1—1 delamination (located in top right) corner and 2 impacts (located central and right side edge) Figure 2.
- D2—4 impacts in each corner of the panel, Figure 3.

Delaminations were induced by adding 2 square layers (25 mm × 25 mm) Polytetrafluoroethylene (PTFE) release film within the outer skin lay-up to prevent the resin from bonding. Impacts were induced through a tooling hammer; between each impact the damaged section was tested with a tap testing hammer to confirm that the structure had indeed become defective.

### 2.2. Inner Skin Heat Conduction

Thermal energy was applied to the inner skin through 4 silicone heater mats measuring 150 mm × 150 mm. The mats were selected to provide a uniform heating stimulus spatially across the surface at the range of operating temperatures (ambient to 80 ${}^{\circ }$C). Four Resistance temperature detectors (RTDs) were used for monitoring temperature, the feedback with which to modulate the power supply to capture the resultant thermal profile on the outer skin.

### 2.3. Outer Skin Temperature Sensor Array

**Figure 4:**
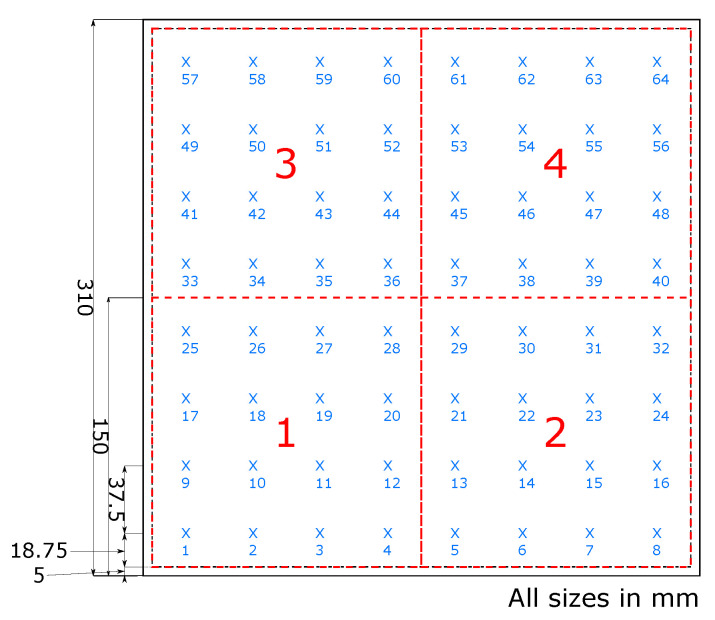
The 8 × 8 RTD airpad array probe locations are shown as blue ’x’, heat zones are shown in red dashed areas.

RTDs measuring 2 mm × 2 mm were used to create an 8 × 8 array of temperature measurement nodes (Figure 4) embedded within a flexible rubber panel measuring 300 mm × 300 mm in dimension. The RTDs were spaced at intervals of 37.5 mm, resulting in a 64 nodal grid across the panel under inspection; each RTD had a 2 pin header to connect jumper wires.

**Figure 5:**
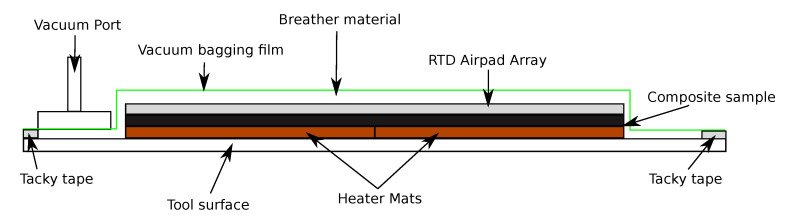
Resistance temperature detector (RTD), sample and heater mat experimental set up within vacuum bagging.

The sensor array captures the temperature profile spatially across the sample panels. Both the inner skin heater mats and outer skin temperature arrays were held in contact with the sample panels using atmospheric vacuum pressure. This was achieved through use of an industry standard vacuum bagging set up commonly used of curing of composite repairs within an MRO. Figure 5 shows the sample sandwiched between the heater mats and RTD Airpad Array tool upon an aluminium tooling surface. The experimental setup was then covered with a porous breather material to provide an airpath to the vacuum port. Once in place the entire experimental set up was sealed using a border of tacky tape and a layer of vacuum bagging film. A vacuum was applied and the set up was inspected for leaks within the bagging film and the tacky tape sealant. When no leaks were found the sample was ready to undergo the step heating.

### 2.4. Control and Data Acquisition

**Figure 6:**
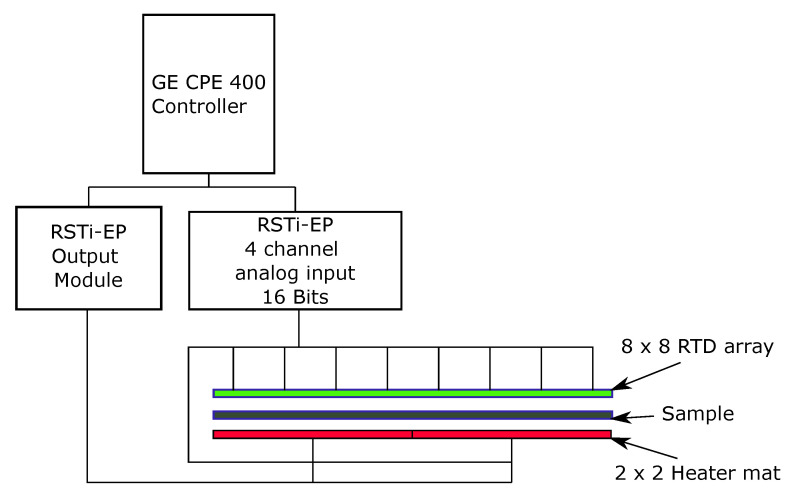
Experimental set up of temperature controller and data acquisition.

A GE Automation CPE400 controller was used to control and record the 68 RTD inputs and 4 digital switch outputs. Figure 6 displays the experimental set up for the RSTi-EP output module used to control the 4 heat zones in a 2 × 2 heater mat arrangement. A set of experiments were conducted to heuristically derive the optimal Proportional-Integral Derivative (PID) constants for the controller to each of the 4 heater zones. 16 RSTi-EP, 4 channel analog inputs where used to acquire temperature measurements from the 64 P100 RTDs on the outer skin and a further RSTi-EP, 4 channel analog input was used to control the heater mats applied to the inner skin.

## 3. Experimental Results

**Figure 7:**
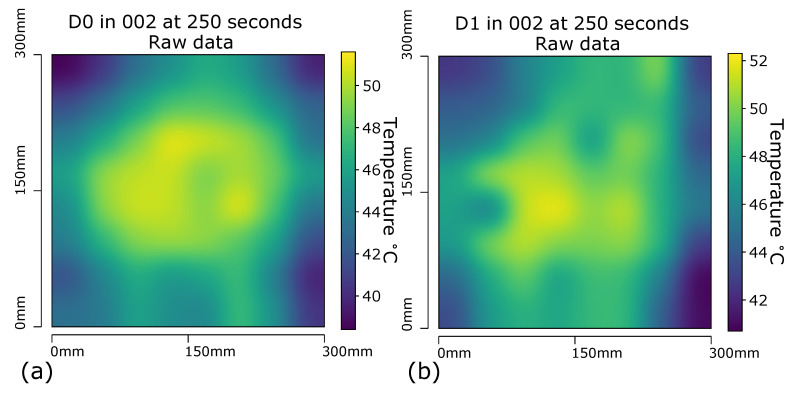
(**a**) Panel D0 at 250 s with Gaussian interpolation applied to RTD array captured data (**b**) Panel D1 at 250 s with Gaussian interpolation applied to RTD array captured data.

Each experimental phase consisted of settling the inner skin temperature of a given sample (through the 4 heater mats) at 20 ${}^{\circ }$C for 15 s and then inducing an immediate temperature step to 80 ${}^{\circ }$C then held for 585 s (total experiment time of 10 min). The 64 RTDs on the outer skin acquired measurements at 1 Hz sampling frequency, with all temperature values logged by timestamps. The visualisation of two examples of the temperature data captured is shown in Figure 7. Figure 7a displays the raw temperature data at 250 s for sample D0 in run 002, and Figure 7b displays the raw temperature data for sample D1 at the same time (250 s).

A Gaussian filter was utilised, a 2-D convolution operation that takes into account the values of temperature surrounding the target zone, in order to generate an approximation of the temperature between the probe points. This also had the the beneficial effect of producing images which would be familiar to operators who had experience with other image based inspection methods, such as IR thermography. On inspection of both Figure 7a,b, a warmer region within the centre of the samples that appears to cool as it approaches the edges of the sample is evident. The cooling is most apparent at the corners of the sample; it is known that the larger an area of a heated body, the quicker the rate of heat transfer (*Q*), captured in the formula for heat transfer;

(2) Q=UAΔT

The edges of the sample have an increased *Q* value as a larger surface area is exposed to ambient temperatures. The behaviour is most marked where edges meet at the corners of the sample as the largest surface area for heat transfer exists at these points.The identification of defects is predicate on identifying ‘cold spots’ within the 64 RTD array on the outer skin, their presence causes a slower rate of thermal conductivity when compared to non-defective regions in close proximity. The initial phase was central to determining of a filtering method that interpolates the 64 discrete temperature values into a continuous thermal map for the outer skin. When viewing these two images it is not immediately obvious where, if any, defects are present. Both images display hot and cold regions, the viewer would have to have knowledge of what a defect free sample’s report should look like in order to assess if the sample is free of defects. Such knowledge of quality samples would require training and experience, and as such using the data in this raw form is not beneficial as a report to a non expert. In order to remove the requirement of a human already having knowledge of a quality part, and the training time involved in this, a subsequent stage was to create a set of baselines of the Gaussian temperature profile for each given time interval according the baseline sample D0 (which does not contain any defects), executed through the experimental capture of the thermal data of D0 under the same conditions as the defect sample D1. The comparison of the defect sample data to the baseline sample data was executed through creating a ratio of each corresponding probe point of the sample under testing against that of the known quality baseline sample.

(3) $$\mathrm{Defect}\;\mathrm{Index}=\frac{\mathrm{Defect}\;\mathrm{Sample}\;\mathrm{Temperature}}{\mathrm{Baseline}\;\mathrm{Sample}\;\mathrm{Temperature}}$$

Plotting the resultant ratio of each point in a quality sample should produce a ’flattened’ image where each probe point ratio is ≈1. Any ratios significantly varying from the value 1 would indicate an areas of interest, i.e., defects, and as such can be quickly identified. A perturbation of the thermal conductivity due to non-uniform materials properties within the sandwich structure are expected for any temperature profile, but these produce outputs manifest within a relatively limited dynamic range. Defects create a much larger divergence.

### 3.1. Panel D1

**Figure 8:**
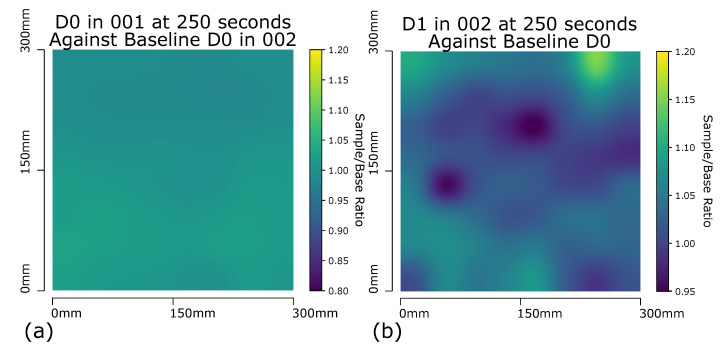
(**a**) Panel D0 run 001 at 250 s with Gaussian interpolation applied to RTD array captured data, each probe compared against corresponding baseline probe on D0 sample run 002. (**b**) Panel D1 at 250 s with Gaussian interpolation applied to RTD array captured data. Each probe compared against corresponding baseline probe on D0 sample at same time and temperature.

**Figure 9:**
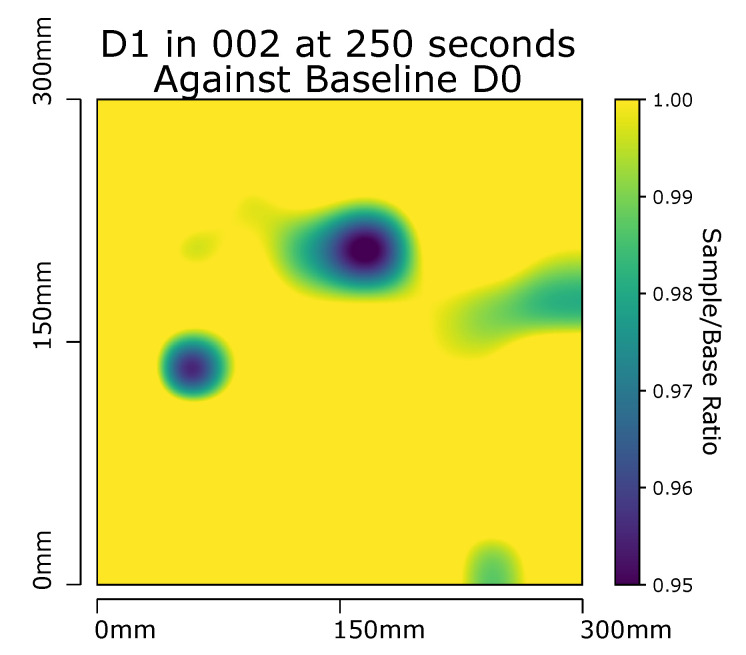
Panel D1 at 250 s with Gaussian interpolation applied to RTD array captured data. Each probe compared against corresponding baseline probe on D0 sample at same time and temperature. Max scale set to 1, min scale set to 0.95 (Sample/Base ratio).

An example of the detection method for impacts is shown in Figure 8a on a the D0 sample when compared against itself at the same point in time during a separate experimental set up. This, unsurprisingly, produces an almost flat image with no obvious areas which are warmer, or cooler than expected. To confirm that the filtering method is able to detect the impact damage of D1 (Figure 8b), it too is compared against a D0 sample run and in this case displays two much darker areas. As impact damage to composite sandwich structures results in a reduction in the thermal conductivity through the sample, the damage manifest as a ’cold spot’ when compared to an undamaged area. These cool spots match the location of the known impact damage induced in sample D1 as seen in Figure 2. A significant cold spot would return a ratio value considerably less than 1 on comparison of the test sample to the known quality baseline sample Equation (3). An additional filter displaying the results within a ratio range between 0.95 and 1 displays quality areas in yellow and areas of interest in blue; shades of green further highlight/classify areas of interest, (Figure 9).

It should also be noted that the delamination within sample D1 is not highlighted with either filter, even though this form of damage is outside the normal variations expected within the structure. However thermal energy is constantly being introduced to the delamination as a consequence of the capture method’s need to capture the temperature profile of the sample during both the ramp-up and steady state heating phases. While delaminations within laminate structures do introduce thermal resistance, this is concentrated at the boundary of the delamination and the homogeneous structure, resulting in the retention of heat for a measurably longer time within the edges of the delamination when compared to that of the surrounding non-delaminated structure during cooling which in turn yields a measurable contrast [23,24,25]. As such, this class of damage is best detected during the rapid introduction and removal of a heat source on the laminate surface. The experimental methodology used within the development makes use of heat mats on the back surface of the sandwich structure in a transmission set up. Thus, in order to detect areas of delaminations, the heat mats would require swift physical application and removal in a reflection set up for the sandwich structure. Such application and removal is not possible within the time restraints required whilst the sample and test equipment are held together via a vacuum bag set-up.

### 3.2. Panel D2

**Figure 10:**
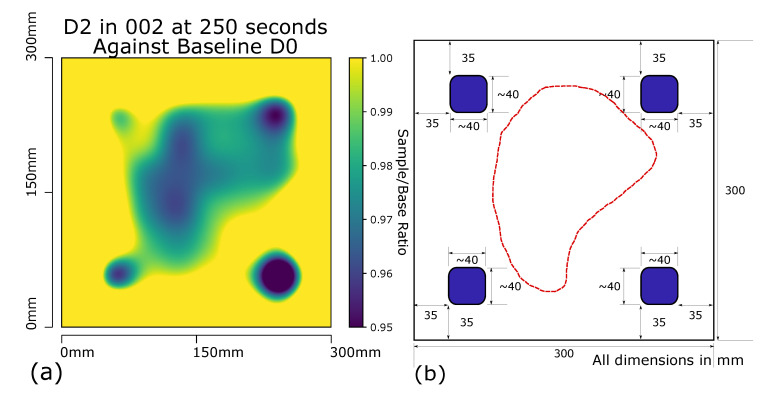
(**a**) Panel D2 at 250 s with Gaussian interpolation applied to RTD array captured data. Each probe compared against corresponding baseline probe on D0 sample at same time and temperature. Max scale set to 1, min scale set to 0.95. (**b**) Panel D2 defect locations. Impact damage blue, induced dis-bond between core and laminate area red dash.

As verification of this methodology to indicate impact induced defects the sample D2 underwent the same experimental capture method as sample D1. Over 4 separate runs of D2 using the same step heating profile and RTD array tool, producing a report as seen in Figure 10a. The 4 points of impact that were induced to D2 can be seen in the report, as well as a larger triangle area between the top two and bottom left impact points. The sample D7 was then inspected by an MRO aerospace engineer using traditional tap inspection methods. The 4 known induced impacts were identified as well as a large area of dis-bond (shown in the red dashed area of Figure 10b) which when compared to the results of the detection method in Figure 10a show a strong correlation. It was discovered that a dis-bond had developed within the sample D2 during the introduction of the 4 impacts.

## 4. Conclusions

The results of the study provide initial evidence that prove the feasibility of a low temperature non-destructive inspection for aerospace sandwich structures. The method does not rely on line-of-sight, obviating the noise due to reflectiveness of the surface under inspection which can be present in the more established IR transmission thermography. The solution utilises commercial-off-the-shelf low cost temperature sensors and heat sources/mats as the basis for the identification of differences in thermal conductivity profiles of composites from low resolution data capture. The system identifies defects through ’cold spots’ owing to the lower thermal conductivity of damaged regions.

The data acquisition and control system applies a stepped temperature profile to the inner skin of test sandwich panels. Defects are identified on both test samples using a 2-D Gaussian filter with baseline probe comparison per sample time stamp. During the validation phase with baseline sample D0 and defect sample D1, the inspection of a second defect panel D2 identified a previously unknown defect, which was subsequently confirmed via a traditional industry standard inspection method. The experimental results show that the method identifies defects according to Fourier’s law of thermal conduction, and as such, only defects inducing a sufficient perturbation to the thermal conductivity of the samples in the direction of transmission are highlighted.

The innovation yields significant benefits to the Aerospace MRO industry; it is amenable to ready integration within an existing practice and thus has a positive benefit on to the processing time of the component, optimising operator effort as well as reducing the risk of extending existing damage through thermal expansion. Further, the method eases the production of rapid reports, image based where areas of interest can be visually inspected to the operator. Through the utilisation of contact temperature sensors, the method can be used in conjunction with repair methods and infrastructure currently incompatible with traditional IR thermography where an unobstructed line-of-sight to the structure surface is required. As the method relies on a baseline temperature of a known quality structure in order to produce the Sample/Base ratio value, it does however require component specific calibration in order to present information of value.

Further investigation is required into the characterisation of delaminations within composite laminates through rapid heat source removal or through the definition of a heating profile capable of indicating delaminations.

## Abbreviations

The following abbreviations are used in this manuscript:

BVID	Barely Visible Impact Damage
CFRP	Carbon Fibre Reinforced Polymer
EASA	European union Aviation Safety Agency
IR	Infra Red
MRO	Maintenance Repair and Overhaul
NDI	Non Destructive Inspection
OEM	Original Equipment Manufacturer
PID	Proportional-Integration Derivative
PTFE	Polytetrafluoroethylene
RTD	Resistance Temperature Detector

## References

[1] Spreen W. (2019). Aerospace maintenance, repair, and overhaul. The Aerospace Business.

[2] Uhlmann E., Bilz M., Baumgarten J. (2013). MRO—Challenge and Chance for Sustainable Enterprises.

[3] Ayeni P., Baines T., Lightfoot H., Ball P. (2011). State-of-the-art of ‘Lean’ in the aviation maintenance, repair, and overhaul industry. Proc. Inst. Mech. Eng. Part B J. Eng. Manuf..

[4] Herrmann A.S., Zahlen P.C., Zuardy I. (2005). Sandwich Structures Technology in Commercial Aviation. Sandwich Structures 7: Advancing with Sandwich Structures and Materials.

[5] Schubel P.M., Luo J.J., Daniel I.M. (2005). Low velocity impact behavior of composite sandwich panels. Composites Part A: Applied Science and Manufacturing.

[6] Schubel P.M., Luo J.J., Daniel I.M. (2007). Impact and post impact behavior of composite sandwich panels. Compos. Part A Appl. Sci. Manuf..

[7] Sun X.C., Hallett S.R. (2017). Barely visible impact damage in scaled composite laminates: Experiments and numerical simulations. Int. J. Impact Eng..

[8] Voto G., Sequeira L., Skordos A.A. Heating rate limits in fast cure processing of thick carbon fibre laminates. Proceedings of the ECCM—18th European Conference on Composite Materials.

[9] Lee J., Soutis C. (2007). A study on the compressive strength of thick carbon fibre-epoxy laminates. Compos. Sci. Technol..

[10] Boey F., Lye S. (1992). Void reduction in autoclave processing of thermoset composites. Composites.

[11] Koushyar H., Alavi-Soltani S., Minaie B., Violette M. (2012). Effects of variation in autoclave pressure, temperature, and vacuum-application time on porosity and mechanical properties of a carbon fiber/epoxy composite. J. Compos. Mater..

[12] Dong C., Zhou J., Ji X., Yin Y., Shen X. (2019). Study of the curing process of carbon fiber reinforced resin matrix composites in autoclave processing. Procedia Manuf..

[13] Luo R., Liu T., Li J., Zhang H., Chen Z., Tian G. (2004). Thermophysical properties of carbon/carbon composites and physical mechanism of thermal expansion and thermal conductivity. Carbon.

[14] Frostig Y., Thomsen O.T. (2008). Non-linear thermal response of sandwich panels with a flexible core and temperature dependent mechanical properties. Compos. Part B Eng..

[15] UK National Aerospace NDT Board (2015). UK Nandtb 18 NDT Method or Technique-Training and Certification Guidance.

[16] Civil Aviation Authority (2017). Safety and Airspace Regulation Group Mandatory Requirements for Airworthiness.

[17] EASA (2017). Disbond of Sandwich Structures.

[18] Cawley P., Adams R. (1988). The mechanics of the coin-tap method of non-destructive testing. J. Sound Vib..

[19] Vavilov V. (2014). Thermal NDT: Historical milestones, state-of-the-art and trends. Quant. InfraRed Thermogr. J..

[20] Georges M., Srajbr C., Menner P., Koch J., Dillenz A. (2018). Thermography and Shearography Inspection of Composite Hybrid Sandwich Structure Made of CFRP and GFRP Core and Titanium Skins. Proceedings.

[21] Ibarra-Castanedo C., Piau J.M., Guilbert S., Avdelidis N., Genest M., Bendada A., Maldague X.P. (2009). Comparative study of active thermography techniques for the nondestructive evaluation of honeycomb structures. Res. Nondestruct. Eval..

[22] Gillespie D.I., Hamilton A.W., McKay E.J., Neilson B., Atkinson R.C., Andonovic I., Tachtatzis C. (2020). Non-Destructive Identification of Fibre Orientation in Multi-Ply Biaxial Laminates Using Contact Temperature Sensors. Sensors.

[23] Maierhofer C., Myrach P., Reischel M., Steinfurth H., Röllig M., Kunert M. (2014). Characterizing damage in CFRP structures using flash thermography in reflection and transmission configurations. Compos. Part B Eng..

[24] Yi Q., Tian G.Y., Malekmohammadi H., Zhu J., Laureti S., Ricci M. (2019). New features for delamination depth evaluation in carbon fiber reinforced plastic materials using eddy current pulse-compression thermography. NDT E Int..

[25] Askaripour K., Zak A. (2019). A Survey of Scrutinizing Delaminated Composites via Various Categories of Sensing Apparatus. J. Compos. Sci..

